# Civilization and Longevity

**Published:** 1876-12

**Authors:** 


### CIVILIZATION AND LONGEVITY.

NATIONS are prolific according to their degradation; as witness the teeming population of China, of India, and of interior Africa. When the Israelites had to work hard, and make brick, getting straw where they could, their numbers increased with great rapidity. The slaves of our own country have more children than their masters. From these facts it is clear, that moral degradation and severe physical labor, each largely increase the number of births.

But civilization presents a paradox. As social amelioration and domestic comforts have made large progress, the average term of human life has been strikingly increased, in that one person died yearly out of every thirty in the last century; while twenty-five years ago, it was found in the same great European States, England, France and Germany, that only one in thirty eight died annually. The present estimate is one out of forty.

At the same time, as civilization advances, the births decrease. Hence, as we progress in a rational civilization, human life is less doubtful, and the chances of its extension steadily increases. Hence with fewer births now than a hundred years ago, among the same number of persons, population is increasing in the more civilized countries, because people live longer in consequence of the social ameliorations of those countries. In the same direction looks the official announcement of M. Villerme, secretary of the poor law commissioners for Havre, that the average age of the rich was twelve years greater than that of the poor. The practical inference is this, that living comfortably is a means of avoiding sickness and of living long. The sooner, therefore, that we attain this end, of living in comfort, the better; while the speediest method of accomplishing it, is for all newly married persons to begin life by the practice of rigid economies, by the exercise and indulgence of plain tastes, and entertaining a manly contempt of the opinion of others as to their style of living, as long as it does not degenerate into meanness—the expenditures being largely within the earnings—giving promise of an age of abundance, of ease and elevation.

